# Supplementary material for: Two Novel Microbacterium Species Isolated from Citrullus colocynthis L. (Cucurbitaceae), a Medicinal Plant from Arid Environments
Source: Microorganisms. 2025 Nov 12;13(11):2583. doi: 10.3390/microorganisms13112583 (PMC12654167; doi:10.3390/microorganisms13112583)
Supplement: Supplementary file 1 [file microorganisms-13-02583-s001.zip › microorganisms-3939124-supplementary.pdf]

Supplementary Information

Table S1. Main characteristics of the draft genome assemblies of Microbacterium isolates from *Citrullus colocynthis*.

**Strain**	**Bacterial Species**	**Assembler**	**Largest Contig**	**Contigs**	**N50**	**Genome Length**	**GC (%)**	**Completeness (%)**	**Contamination (%)**	**Depth (X)**	**Protein coding sequence (CDS)**	**tRNA aa**	**16S Ribosomal RNA (%)**	**Accession number (SRA)**
AGC47	*M. xerophyticum*	MaSuRCA	902,967	17	512,876	4,072,017	67.24	99.19	1.06	209	3,971	20	59.8	SRR29855773
AGC62	*M. umsixpiens*	MaSuRCA	83,195	257	25,449	3,913,100	68.54	99.17	1.01	47	3,996	20	95.5	SRR29855759
AGC85	*M. xerophyticum*	MaSuRCA	1,163,575	9	988,508	4,069,068	67.23	97.47	0.61	522	3,932	20	96.7	SRR29855758

Table S2. Taxonomic identification of Novel *Microbacterium* species from *Citrullus colocynthis* based on 16S rRNA gene sequencing using the Sanger method.

**Strain**	**Origin**	**Species**	***length (bp)***	***Identity (%)***	***Coverage (%)***	***Accession number***
AGC62	Cc-F	*Microbacterium sp.*	604	100	100	PV706301
AGC85	Cc-R	*Microbacterium sp.*	684	99.51	100	PV706316

Table S3. Gene count for major functional gene categories involved in chemotaxonomic characterization.

**Functional category**	**Gene**	**AGC47**	**AGC62**	**AGC85**
**Cell_wall_sugars**	D-glycero-alpha-D-manno-heptose 1-phosphate guanylyltransferase	1	1	1
	D-glycero-alpha-D-manno-heptose-7-phosphate kinase	1	1	1
	GDP-L-fucose synthase	1	1	1
	GDPmannose 4,6-dehydratase	1	1	1
	UDP-galactopyranose mutase	1	1	1
	UDP-glucose 4-epimerase	2	2	2
	UDP-glucuronate 4-epimerase	1	1	1
	UDPglucose 6-dehydrogenase	1	1	1
	UTP--glucose-1-phosphate uridylyltransferase	1	1	1
	dTDP-L-rhamnose 4-epimerase	1	1	1
	mannose-1-phosphate guanylyltransferase	1	1	1
	mannose-6-phosphate isomerase	1	1	1
	phosphomannomutase	1	1	1
**FAME_support_optional**	2-isopropylmalate synthase	1	1	1
	3-isopropylmalate dehydratase	1	1	1
	3-isopropylmalate dehydrogenase	1	1	1
	3-isopropylmalate/(R)-2-methylmalate dehydratase small subunit	1	1	1
	L-serine/L-threonine ammonia-lyase	1	1	1
	leucine dehydrogenase	1	1	1
	threonine dehydratase	1	1	1
**MK_ring_men**	1,4-dihydroxy-2-naphthoyl-CoA hydrolase	2	2	2
	2-methoxy-6-polyprenyl-1,4-benzoquinol methylase	1	1	1
	2-succinyl-5-enolpyruvyl-6-hydroxy-3-cyclohexene-1-carboxylate synthase	1	1	1
	2-succinyl-6-hydroxy-2,4-cyclohexadiene-1-carboxylate synthase	1	1	1
	chorismate lyase / 3-hydroxybenzoate synthase	1	1	1
	chorismate lyase	1	0	1
	demethylmenaquinone methyltransferase / 2-methoxy-6-polyprenyl-1,4-benzoquinol methylase	1	1	1
	isochorismate synthase / 2-succinyl-5-enolpyruvyl-6-hydroxy-3-cyclohexene-1-carboxylate synthase / 2-succinyl-6-hydroxy-2,4-cyclohexadiene-1-carboxylate synthase / o-succinylbenzoate synthase	1	1	1
	naphthoate synthase	1	1	1
	o-succinylbenzoate synthase	1	1	1
	o-succinylbenzoate---CoA ligase	2	2	2
	polyprenyldihydroxybenzoate methyltransferase / 3-demethylubiquinol 3-O-methyltransferase	1	1	1

	adenosylhomocysteine/aminodeoxyfutalosine nucleosidase	1	1	1
	aminodeoxyfutalosine deaminase	2	2	2
	cyclic dehypoxanthinyl futalosine synthase	1	1	1
	futalosine hydrolase	1	1	1
**PG_ligases_DAA**	N-acetylornithine carbamoyltransferase	1	1	1
	UDP-N-acetylmuramoyl-L-alanine---L-glutamate ligase	1	1	1
	arginine deiminase	1	1	1
	lipid II isoglutaminyl synthase (glutamine-hydrolysing)	2	2	2
	ornithine carbamoyltransferase	1	1	1
	undecaprenol kinase	1	1	1
**Polar_lipids_Teichoic**	CDP-glycerol glycerophosphotransferase [	1	1	1
	CDP-ribitol ribitolphosphotransferase / teichoic acid ribitol-phosphate polymerase [	1	1	1
	choline kinase	1	0	1
	choline-phosphate cytidylyltransferase	1	1	1
	lipoteichoic acid ligase	1	1	1
	lipoteichoic acid primase	1	0	1
	polyisoprenyl-teichoic acid--peptidoglycan teichoic acid transferase	1	1	1
	teichoic acid glycerol-phosphate primase	1	1	1
	teichoic acid glycerol-phosphate transferase	1	1	1
**Prenyl_chain**	teichoic acid ribitol-phosphate primase	1	1	1
	(2Z,6Z)-farnesyl diphosphate synthase	1	1	1
	hexaprenyl diphosphate synthase	1	1	1
	hexaprenyl-diphosphate synthase large subunit	1	1	1
	isopentenyl phosphate kinase	1	1	1
	isopentenyl-diphosphate Delta-isomerase	1	1	1
	trans,polycis-decaprenyl diphosphate synthase	1	1	1
	trans,polycis-polyprenyl diphosphate synthase	1	1	1
	tritrans,polycis-undecaprenyl-diphosphate synthase [geranylgeranyl-diphosphate specific]	1	1	1

Table S4. Phenotypic profile and carbon-source utilization of type strains *Microbacterium umsixpiens AGC62* and *Microbacterium xerophyticum AGC85* versus their colosest relatives' reference type strains.

	Microbacterium umsixpiens		Microbacterium xerophyticum	
	Microbacterium umsixpiens AGC62	Microbacterium liquefaciens DSM 20638	Microbacterium xerophyticum AGC85	Microbacterium profundi Shh49
ANI	86.5%		89.1%	
dDDH	44.4%		33%	
Temperature		30 °C		30 °C
pH	5-6	4-10	6-7.5	6.0–9.5
NaCl	0-8%	0-7 %	0-4%	0-4 %
Oxygen tolerance	Aerobe	Aerobe	Aerobe	Aerobe
Gram staining	Gram positive	Gram positive	Gram positive	Gram positive
colour	egg-yellow	Yellow to yellow orange	Neon yellow	Yellow
shape	Round/ convex	Round/ convex	Round/ pulvinate	Round
Texture	viscoid	smooth	smooth	-
size	2 mm	1–3 mm	2 mm	-
substrate	Dextrin; D-maltose; D-trehalose; D-cellobiose; gentiobiose; sucrose; D-turanose; D-raffinose; D-melibiose; β-methyl-D-glucoside; D-salicin; N-acetyl-D-glucosamine; N-acetyl-β-D-mannosamine; N-acetyl-D-galactosamine; α-D-glucose; D-mannose; D-fructose; D-galactose; 3-	D-glucose, D-mannitol, D-ribose, D-xylose, esculin, gelatin, glycogen, lactose, maltose, nitrate	D-fucose, L-fucose, L-rhamnose, sodium lactate,	Acetate, arabinose, arginine, aspartate, cellobiose, esculin, fructose, galactose, glucose, glycerol.

	methyl-glucose; D-fucose; L-fucose; L-rhamnose; inosine.			
